# Comprehensive human respiratory genome catalogue underlies the high resolution and precision of the respiratory microbiome

**DOI:** 10.1093/bib/bbae620

**Published:** 2024-11-24

**Authors:** Yinhu Li, Guangze Pan, Shuai Wang, Zhengtu Li, Ru Yang, Yiqi Jiang, Yu Chen, Shuai Cheng Li, Bairong Shen

**Affiliations:** Institutes for Systems Genetics, Frontiers Science Center for Disease-related Molecular Network, West China Hospital, Sichuan University, No. 2222 Xinchuan Road, Gaoxin District, Chengdu 610212, China; Chinese Academy of Sciences Key Laboratory of Brain Connectome and Manipulation, Shenzhen Key Laboratory of Translational Research for Brain Diseases, The Brain Cognition and Brain Disease Institute, Shenzhen Institute of Advanced Technology, Chinese Academy of Sciences, Shenzhen–Hong Kong Institute of Brain Science–Shenzhen Fundamental Research Institutions, No. 1068 Xueyuan Avenue, Nanshan District, Shenzhen 518055, China; Department of Computer Science, City University of Hong Kong, 83 Tat Chee Avenue, Kowloon Tong, Hong Kong 999077, China; Department of Computer Science, City University of Hong Kong, 83 Tat Chee Avenue, Kowloon Tong, Hong Kong 999077, China; State Key Laboratory of Respiratory Disease, National Clinical Research Center for Respiratory Disease, Guangzhou Institute of Respiratory Health, the First Affiliated Hospital of Guangzhou Medical University, No. 1 Kangda Road, Haizhu District, Guangzhou 510120, China; Department of Neonatology Nursing, West China Second University Hospital, West China School of Nursing, Sichuan University, No. 1416 Chenglong Avenue, Jinjiang District, Chengdu 610041, China; Department of Computer Science, City University of Hong Kong, 83 Tat Chee Avenue, Kowloon Tong, Hong Kong 999077, China; Chinese Academy of Sciences Key Laboratory of Brain Connectome and Manipulation, Shenzhen Key Laboratory of Translational Research for Brain Diseases, The Brain Cognition and Brain Disease Institute, Shenzhen Institute of Advanced Technology, Chinese Academy of Sciences, Shenzhen–Hong Kong Institute of Brain Science–Shenzhen Fundamental Research Institutions, No. 1068 Xueyuan Avenue, Nanshan District, Shenzhen 518055, China; Department of Computer Science, City University of Hong Kong, 83 Tat Chee Avenue, Kowloon Tong, Hong Kong 999077, China; Institutes for Systems Genetics, Frontiers Science Center for Disease-related Molecular Network, West China Hospital, Sichuan University, No. 2222 Xinchuan Road, Gaoxin District, Chengdu 610212, China

**Keywords:** human respiratory genome catalogue, respiratory microbiome, metagenomic assembly, microbial etiology

## Abstract

The human respiratory microbiome plays a crucial role in respiratory health, but there is no comprehensive respiratory genome catalogue (RGC) for studying the microbiome. In this study, we collected whole-metagenome shotgun sequencing data from 4067 samples and sequenced long reads of 124 samples, yielding 9.08 and 0.42 Tbp of short- and long-read data, respectively. By submitting these data with a novel assembly algorithm, we obtained a comprehensive human RGC. This high-quality RGC contains 190,443 contigs over 1 kbps and an N50 length exceeding 13 kbps; it comprises 159 high-quality and 393 medium-quality genomes, including 117 previously uncharacterized respiratory bacteria. Moreover, the RGC contains 209 respiratory-specific species not captured by the unified human gastrointestinal genome. Using the RGC, we revisited a study on a pediatric pneumonia dataset and identified 17 pneumonia-specific respiratory pathogens, reversing an inaccurate etiological conclusion due to the previous incomplete reference. Furthermore, we applied the RGC to the data of 62 participants with a clinical diagnosis of infection. Compared to the Nucleotide database, the RGC yielded greater specificity (0 versus 0.444, respectively) and sensitivity (0.852 versus 0.881, respectively), suggesting that the RGC provides superior sensitivity and specificity for the clinical diagnosis of respiratory diseases.

## Introduction

Extensive studies unequivocally demonstrate that the widespread microbes within the human respiratory tract play roles in preserving respiratory health [1–4]. Specifically, the commensal microbiota that inhabits the upper respiratory tract is resistant to microbial colonization of the mucosal surfaces by pathogenic microorganisms [5]. Recent studies conducted during the COVID-19 pandemic suggest that the respiratory microbiome (RM) influences the severity of COVID-19 and is associated with secondary respiratory infections [6, 7]. Furthermore, the symbiotic respiratory microbiota synergistically collaborates with the immune system of the host to thwart microbial intrusion [8–10]. For instance, in conjunction with *Haemophilus influenzae*, the RM augments the proficiency of neutrophils in eliminating *Streptococcus pneumoniae* [10]. Microbial genomes provide a fundamental basis for accurately understanding the composition and functionality of the RM [11]. For example, in a previous study, decoded microbial genomes facilitated the identification of specific microbial genes involved in trimethylamine-*N*-oxide biosynthesis, allowing the investigation of host–microbiome relationships [12].

Constructing a reference genome for the respiratory microbial species is crucial to understand the RM. However, given the challenges of capturing the full diversity of the RM by microbial cultivation, utilizing metagenomic data to reconstruct metagenome-assembled genomes (MAGs) is pivotal to establishing comprehensive respiratory microbial genome references, thus enhancing the precision of microbiome analysis [13–16]. Our previous work constructed the Respiratory Microbial Gene Catalogue (RMGC) using metagenomic data from 334 respiratory samples [16]. Owing to the inherent limitations of sample size, repetitive elements within microbial genomes, and short-read sequencing, the RMGC encompassed only 125 co-abundance gene groups, thus failing to capture the full spectrum of microorganisms that inhabit the respiratory tract [16, 17]. These inadequately assembled and unrepresentative respiratory genomes within the RMGC pose challenges to comprehensively understanding respiratory microbes. This highlights the challenges of establishing a representative reference of the respiratory microbial genome.

Therefore, in this study, we constructed a representative respiratory genome catalogue (RGC) by integrating large datasets and better assembly algorithms to enable high-resolution annotation of the human respiratory genome. In addition to encompassing a wide range of respiratory microorganisms, the RGC also prioritizes enhancing assembly continuity and optimizing binning performance to obtain high-quality MAGs. Recent studies demonstrate that single-molecule sequencing improves the continuity of metagenomic assembly by capitalizing on longer read lengths [18–21]. For example, Moss et al. used Nanopore sequencing to reconstruct microbial genomes from metagenomic data, achieving greater assembly continuity and enabling the recovery of 20 complete bacterial genomes using MinION [19]. Furthermore, studies have combined Nanopore data with next-generation sequencing data to address sequencing errors introduced by long-read sequencing [20, 21]. Here, we integrated next-generation sequencing and single-molecule sequencing data, developed a metagenomic assembly algorithm, and constructed a highly contiguous and representative RGC.

Accordingly, we performed large-scale MAG construction to establish a comprehensive RGC by using 0.42 and 9.08 Tbp of long- and short-read data, respectively, obtained from 4191 respiratory samples. Adhering to the median criteria of the Minimum Information about a Metagenome-Assembled Genome (MIMAG) standards [22], the RGC comprises 551 non-redundant bacterial genomes and 1 archaeal genome. Facilitated by improved species and functional profiles within the RGC, we revisited a previous pneumonia RM project and identified pneumonia-specific respiratory pathogens. Accordingly, compared to the NCBI Nucleotide (NT) database, the RGC yielded greater specificity and sensitivity in a cohort of 62 participants with a clinical diagnosis of infection. Thus, the RGC will be a valuable resource for future investigations of the human respiratory microbiota.

## Materials and methods

### Ethics

This study followed the principles outlined in the Declaration of Helsinki and received approval from the ethics committees of the First Affiliated Hospital of Guangzhou Medical University (ethical number: 2020–36). Prior to their participation, all individuals provided written informed consent and willingly volunteered to undergo investigation for scientific research.

### Illumina data collection

We comprehensively searched for publicly available respiratory metagenomic data on PubMed prior to December 2022 using the following criteria: (i) inclusion of the keywords ‘respiratory metagenomic data’ or ‘respiratory metagenome’; (ii) datasets comprising no fewer than 10 samples; and (iii) projects performed on an Illumina platform. Ultimately, we collected 4067 public data from 20 projects (Supplementary Table 1), providing next-generation sequencing data for subsequent respiratory metagenomic analysis [16, 23–41].

### Participant recruitment and respiratory sample collection

We specifically enrolled 124 participants with suspected respiratory infections from the First Affiliated Hospital of Guangzhou Medical University, Guangzhou, China. We collected respiratory samples from them and performed Nanopore sequencing. We collected sputum, oropharyngeal swabs, and bronchoalveolar lavage fluid (BALF) specimens from the participants using sterile specimen containers. We carefully stored the collected specimens in a liquid nitrogen freezing box and transported them to the biosafety level three laboratory of the Guangdong Centres for Disease Control. We mixed 140 μl respiratory sample with 560 μl AVL solution and then stored them in a refrigerator at −80°C for preservation.

### Nanopore library preparation and sequencing

Following the manufacturer’s protocol, we extracted total DNA from the sputum specimens using an E.Z.N.A. Soil DNA Kit (Omega Bio-Tek, Norcross, United States). We used the extracted DNA samples that met the specified quality criteria to construct Nanopore libraries following the instructions provided in the Ligation Sequencing Kit (Nanopore, Cambridge, United Kingdom). Accordingly, we obtained 124 high-quality DNA libraries and subjected them to metagenomic sequencing using the MinION platform (Nanopore, Cambridge, United Kingdom).

### Illumina data filtration and metagenomic assembly

We employed prinseq++ software (version 1.2) to perform quality filtering on all next-generation sequencing data [42], eliminating sequences with more than 10 low-quality bases (i.e. <Q20). Subsequently, we employed bwa software (version 0.7.17-r1188) with the default parameters to align the high-quality reads to the *Homo sapiens* reference genome (GRCh38) [43]. To remove reads aligned to the human genome, we utilized samtools (version 1.3.1) [44]. Prior to metagenomic assembly, we merged all high-quality filtered paired-end reads within each project. For paired-end reads, we employed the ‘repair.sh’ script from bbmap (version 39.01–0) to sort the reads and eliminate unmated ones [45]. For metagenomic next-generation sequencing data assembly, we predominantly utilized the software and databases integrated within MetaWRAP (version 1.3.2) [46]. We performed preliminary assembly using MEGAHIT (version 1.2.9) with kmer parameters set at 25, 50, and 75, respectively [47]. Subsequently, we conducted initial binning of the assembly results using MetaBAT (version 2.12.1) [48], MaxBin (version 2.0) [49], and CONCOCT (version 1.1.0) [50]. We selected and merged the highest-quality bins from the initial binning results using pplacer (version 1.1.alpha19–0-g807f6f3) [51]. After evaluating the quality of the bins using CheckM (version 1.0.18) [52], we retained the bins with completeness >50% and contamination <10% as representatives for each dataset.

### Optimization of Illumina metagenomic assemblies

To obtain longer sequences, we adopted a metagenomic scaffolding approach based on the contigs assembled using MEGAHIT [47]. This process is inspired by the conjugate graph proposed by Jia et al. [53]. With the contigs, a collection of DNA segment $C=\left\{{C}_1,{C}_2,\dots{C}_n\right\}$ can be constructed. The junction set $J$ among $C$ can be derived from the next-generation sequencing reads alignment. Given a junction $j=<{C}_i,{C}_j>$, a number $w(j)$ represents the weight of this junction, which indicates the reads pair counts that aligned ${C}_i$ and ${C}_j$ separately. According to the conjugate graph definition, each ${C}_i$ can be represented as conjugate vertices ${V}_i+$ and ${V}_j-$, denoting the positive and negative DNA strands, respectively. Similarly, each connection $j$ can be represented as a pair of conjugate edges $<{V}_i+,{V}_j->$ and $<{V}_j-,{V}_i+>$. Based on this conjugate graph derived from assembly contigs, we consider the meta-genome scaffolding problem as finding the maximum weighted path on this graph. Accordingly, we propose a constrained Kuhn–Munkres algorithm to solve this problem. Owing to the shared weights and copy numbers of conjugate elements in the conjugate graph, we introduced a conditional restriction in the original Kuhn–Munkres algorithm. Specifically, when searching for an augmenting path, if an edge is already included in a previous path, its conjugate edge will be excluded from the search.

After scaffolding, for a pair of contigs, ${c}_i$ and ${c}_j$, on the scaffolds’ path, we connected the two contigs initially if the longest common subsequence between their ends could be found. For the remaining contigs, we performed a gap closure procedure on the scaffolds with two steps: (i) gap size estimation and (ii) gap filling.

*Gap size estimation*. We used BWA to align the read sequences with all contigs [43], retaining only the read pairs that aligned with two separate contigs. Let ${R}_{ij}$ be a set of read pairs, where ${read}_1\left({r}_1\right)$ and ${read}_2\left({r}_2\right)$ are aligned with ${c}_i$ and ${c}_j$, respectively. For a read pair (${r}_1$, ${r}_2$) in ${R}_{ij}$, the soft-clip lengths of ${r}_1$ and ${r}_2$ are denoted by ${r}_1(s)$ and ${r}_2(s)$, and the distances from ${r}_1$ and ${r}_2$ to the ends of ${c}_i$ and ${contig}_j$ are denoted by ${c}_i\left({r}_1\right)$ and ${c}_j\left({r}_2\right)$, respectively. When ${r}_1(s)$ is greater than 0, ${c}_i\left({r}_1\right)$ will be 0; the same applies to ${r}_2$. Given the average insertion size (i.e. $isize$) and variance (i.e. $istd$) of the sequencing data, we calculated the gap length supported by ${r}_1$ and ${r}_2$ using the following formula:


$$ \mathrm{Gap}\left({r}_1,{r}_2\right)=\left( isize- len\left({r}_1\right)- len\left({r}_2\right)\right)+\left({r}_1(s)+{r}_2(s)\right)-\left({c}_i\left({r}_1\right)+{c}_j\left({r}_2\right)\right) $$

For ${R}_{ij}$, we only kept read pairs where $\mathrm{abs}\left(\mathrm{Gap}\left({r}_1,{r}_2\right)\right)< isize+ istd$, resulting in the filtered reads set as ${RF}_{ij}$.


$$ \mathrm{Gap}\left({c}_i,{c}_j\right)=\frac{\sum_{\begin{array}{c}r1,r2\ \\{} in\ {RF}_{ij}\end{array}} Gap\left(r1,r2\right)\ }{count\left({RF}_{ij}\right)} $$

*Gap filling*. We merged the unaligned reads and the reads used in the gap size estimation stage. We then reassembled these reads into short contigs using MEGAHIT [47]. With these short contigs, we applied LR_Gapcloser software (version 3) to fill the gaps across the scaffolds, resulting in the final assembled sequences [54].

### Nanopore data filtration and metagenomic assembly

We employed NanoFilt (version 2.8.0) to filter the Nanopore sequencing data, explicitly removing reads shorter than 150 bp and those with an average quality score less than 10 [55]. Subsequently, we used Minimap2 software (version 2.24-r1122) [56] with the default parameters to align the high-quality reads to the *Homo sapiens* reference genome (GRCh38) and utilized samtools (version 1.3.1) to remove reads aligned to the human genome [44]. Following this, we merged all filtered high-quality Nanopore reads and conducted preliminary metagenomic assembly using metaFlye software (version 2.9.1-b1780) [57]. Similarly, we performed initial binning of the assembly results using the MetaBAT2, MaxBin, and CONCOCT methods [48–50]. Next, we merged and selected the best-quality bins from the initial binning results using the pplacer algorithm [51]. After evaluating the quality of the bins using CheckM [52], we retained bins with completeness >50% and contamination <10%.

### Integration of Illumina and Nanopore metagenomic assemblies

To integrate the bins obtained from multiple datasets, we initially incorporated all refined bins into a genome tree using pplacer software [51] and selected the highest-quality bins meeting the criteria of completeness >50% and contamination <10%. Subsequently, to eliminate redundancy within our collection of high-quality genomes, we employed the ‘dereplicate’ module in dRep software (version 3.4.2) to remove duplicate genomes exhibiting a shared average nucleotide identity (ANI) > 95.0% or a shared genome coverage >30% (parameters: ‘-comp 50 -con 10’) [58]. We defined the resulting set of filtered MAGs as the RGC and employed it in subsequent analyses.

### Species-level clustering of MAGs in the RGC

We performed taxonomic classification for genomes in the RGC using the ‘classify_wf’ module implemented in genome taxonomy database (GTDB)-Tk software (version 2.1.1) [59]. Leveraging the GTDB (version 08-RS214) [60], we assigned species-level taxonomy to the MAGs with a shared ANI > 95% with reference genomes. Meanwhile, we designated the MAGs that could not be confidently assigned at the species level as novel species within the RGC database while retaining their genus-level annotations.

### Gene prediction and functional annotation

We employed Prokka software (version 1.14.6) to predict the genes of MAGs with the ‘—metagenome’ parameter [61]. Utilizing the protein sequences from the comparative antibiotic resistance database (CARD, version 3.2.7) [62], we performed blastp (version 2.13.0+) analysis to annotate antibiotic resistance genes (ARGs) with the parameters, ‘-max_target_seqs 1’ and ‘-evalue 1e-5’ [63]. Similarly, to identify virulence genes, we performed blastn analysis (version 2.13.0+) based on nucleotide sequences from the virulence factor database (VFDB, version 26 May 2023) [64]. Furthermore, we used antiSMASH software (version 6.1.1) with the parameter ‘--genefinding tool none’ to identify metabolic gene clusters (MGCs) within the MAG of the RGC [65]. Finally, we utilized BiG-SCAPE software (version 1.1.5) with the default parameters to classify and summarize the metabolic annotation results [66].

### Construction of phylogenetic trees

Using the ‘classify_wf’ module implemented in GTDB-Tk software [59], we generated a ‘user_msa’ file containing the aligned protein sequences of 120 core bacterial genes for all MAGs. Based on the aligned sequences, we employed the ‘infer’ module of GTDB-Tk software to construct maximum likelihood phylogenetic trees. We visualized the resulting trees using Interactive Tree Of Life (version 6.7.5) [67].

### Taxonomical annotation for the RGC and other databases

For the RGC and NT databases, we used Salmon software (version 0.14.1) to perform species annotation [68]. First, we constructed libraries for the RGC and NT databases [69] using the ‘index’ function in Salmon. Next, we employed the ‘quant’ function in Salmon to align the metagenomic data to the databases, enabling quantitative evaluation of the species composition within the samples. Last, we extracted and summarized the species compositions of the samples using the ‘summarize_salmon_files.py’ script in MetaWRAP [46].

For the standard database in Kraken2 and the Unified Human Gastrointestinal Genome (UHGG) database [70], we employed Kraken2 (version 2.1.3) and Bracken (version 2.8) software to perform taxonomical annotation [71]. First, we employed the Kraken2 software to perform taxonomical annotations with the standard or UHGG databases. Second, we utilized Bracken to adjust the taxonomical profiles acquired in the last step. To obtain the optimal adjusted results, we constructed libraries with different read lengths for the databases (i.e. 100, 150, 200, and 250 bp) and selected the appropriate read length according to the metagenomic data of each sample. Finally, we unified all annotation results into the same format using the ‘kreport2mpa.py’ script in Bracken.

Given that the taxonomical abundances calculated by Salmon and Kraken2 were measured in copy number per million reads and aligned reads number, respectively, we converted copy number per million reads into aligned reads number using the following formula to compare taxonomical abundance across different databases:


$$ {R}_i={CPM}_i\ast{L}_i/10000000 $$

${CPM}_i$ is the abundance value calculated by Salmon, ${R}_i$is the reads number aligned to genome *i*, and ${L}_i$ is the length of genome *i*.

### Simulation of metagenomic data

We used an in-house script to simulate RM metagenomic data to assess the accuracy of the RGC, UHGG, and standard database in Kraken2 for taxonomical annotation. For each RM metagenomic dataset, the script would randomly select 10 genomes from the RGC, assign the selected genomes random abundance, and generate 3 Gbp of metagenomic data.

To evaluate the performance of the RGC for the taxonomical annotation of metagenomic data under various read lengths, we reused the metagenomic data from a previous project, which contained 80 metagenomic datasets with 250 paired-end reads. We applied seqkit software (version 2.5.1) to trim the reads and obtained metagenomic data with 100, 150, and 200 paired-end reads for further analysis [72].

### Statistics

After obtaining the taxonomical results for the exemplified datasets, we applied a centered log-ratio transformation for RM normalization before performing other inferential statistics [73]. To assess the recovery of all species in the exemplified samples, we employed the ‘specaccum’ function in R to generate an accumulation curve. We determined the bacterial diversity at the species level by calculating the Shannon index and the observed species number using the ‘vegan’ package in R [74]. To explore the overall features of the RM in the exemplified dataset, we performed principal coordinate analysis on all samples based on Atchison distances using the ‘vegan’ package in R [74]. Subsequently, we filtered taxonomical features present in fewer than three samples and performed differential analysis between the healthy and pneumonia groups using the two-tailed Welch’s *t*-test. We adjusted for multiple statistical tests using the Benjamini–Hochberg method (adjusted *P* < 0.05) and visualized the results using the ‘ggplot2’ package in R.

## Results

### Effective assembly of a high-quality RGC

**Figure 1:**
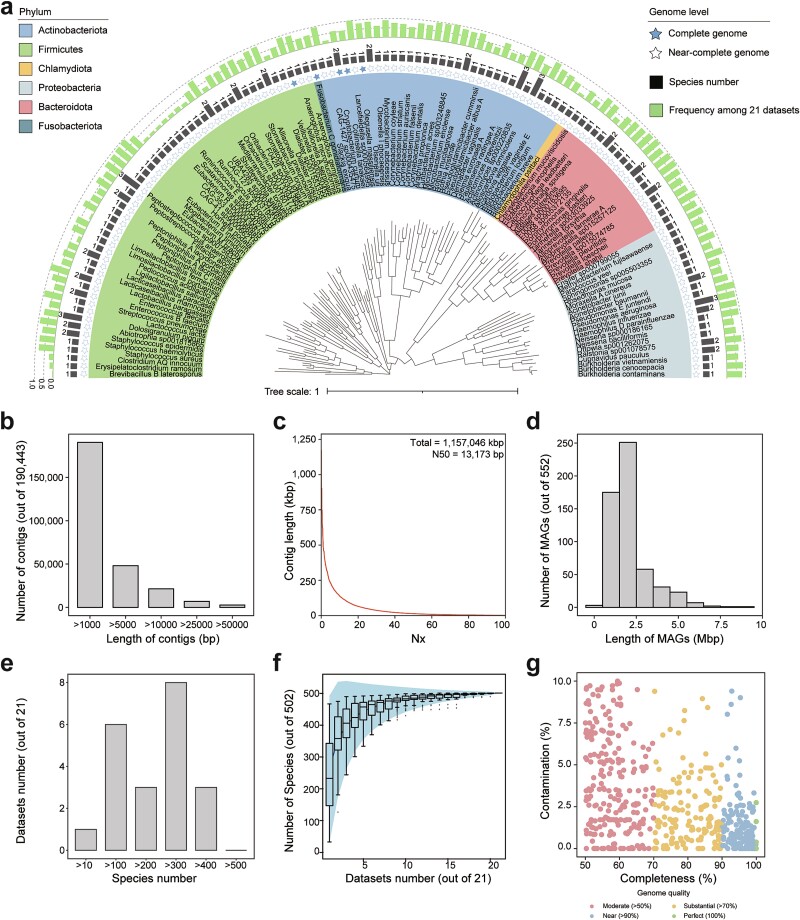
**Effective assembly of the species-level MAGs in the RGC.** (a) Maximum-likelihood phylogenetic tree constructed from 159 high-quality MAGs. Solid stars represent the MAGs with 100% completeness. The bars in the outer first circle represent the number of assembled genomes for each species-level MAG. The bars in the outer second circle represent the frequencies of the MAGs among the 21 datasets. (b) Length distribution of the assembled contigs. The x- and y-axes represent the length and number of contigs, respectively. (c) Contig cumulative curve. The x-axis represents Nx, and the y-axis represents the length of contigs. The N50 of the contigs reached 12,173 bp. (d) Length distribution of the MAGs. The x- and y-axes represent the length and number of MAGs, respectively. (e) Species number distribution among the 21 datasets. The x- and y-axes represent the numbers of species and datasets, respectively. (f) Accumulation curves of the species. Horizontal and vertical coordinates indicate the numbers of datasets and species, respectively. (g) Quality distributions of the MAGs in the RGC. The x- and y-axes represent the completeness and contamination of the MAGs, respectively.

The genomic sequences recovered in the comprehensive RGC represent the microorganisms in the human respiratory tract, thus tackling the challenge posed by the substantial microbes with unknown sources in public databases. In the present study, we curated a comprehensive dataset of 4067 Illumina metagenomic data from 20 independent studies (Supplementary Table 1). We also sequenced 124 respiratory samples with Nanopore technology obtained from healthy individuals and patients with respiratory infections. After stringent data filtering to remove low-quality and host-related sequences, 9.08 Tbps of short-read data and 415.52 Gbps of long-read data remained. We performed metagenomic assembly and assembly binning for each dataset. Subsequently, we used the dRep software [58] package to eliminate duplicate MAGs from the assembled sequences effectively and identify 552 MAGs that passed the median criteria of the MIMAG standards [22] (i.e. >50% completeness and < 10% contamination) (Fig. 1). Within the MAG collection, 159 high-quality genomes showed >90% completeness and < 5% contamination (Fig. 1a, Supplementary Table 2).

In addition to the MAGs, this large-scale assembly of the respiratory metagenome demonstrates favorable assembly continuity. The RGC consists of 190,443 contigs (>1 kbps) with an N50 length of 13 kbps, resulting in a total assembly length of 1157 Mbps (Fig. 1b and1c). The largest contig within the RGC has a length of 1.72 Mbps, with 2681 contigs exceeding 50 kbps in length. Among the MAGs obtained, the average genome length reached 2.096 ± 1.179 Mbps (range: 0.460–8.536 Mbps), including five genomes that achieved 100% completeness (Fig. 1d, g). By annotating the 159 high-quality MAG species, we observed a predominance of the Firmicutes, Actinobacteriota, Bacteroidota, and Proteobacteria phyla (Fig. 1a).

### Representativeness of genomes recovered in the RGC

**Figure 2:**
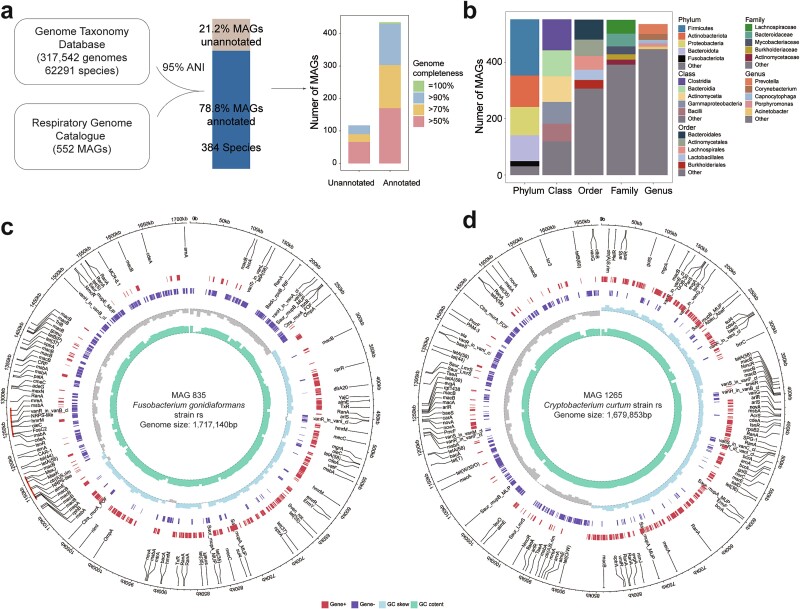
**Representativeness of respiratory microbes using the RGC.** (a) Cluster analysis results of 522 genomes in the RGCs and 317,542 GTDs with a threshold ANI of 95%; 78.8% of the RGC genomes exhibited significant homology (>95% ANI) with the GTDB, while the remaining 21.2% were unique to the RGC dataset. (b) Taxonomic distribution of the RGC dataset at the phylum, class, order, family, and genus levels. Only the top five taxonomic units are shown for each hierarchical level, while the remaining classifications are labelled ‘other’. (c, d) two complete MAGs were recovered in the RGC. The outermost (i.e. first) circle represents the scale (kbp); the second circle displays ARGs in the two currently assembled complete MAGs; the third circle represents genes on the genomic positive strand; the fourth circle represents genes on the genomic negative strand; the fifth circle represents the GC skew; and the sixth circle represents GC content. In plot c, the dashed lines next to the outer first circle represent the MGCs in *Fusobacterium gonidiaformans* strain rs. The centers of the plots display the species information and genome sizes for the two complete MAGs.

The representative genomes in the RGC reveal the presence of previously unidentified species within the RM. We compared the 552 MAGs with dereplicated genomes in the GTDB to assign their taxonomical positions, employing an average nucleotide identity threshold of 95% (Fig. 2a, Supplementary Table 2). Accordingly, 435 MAGs exhibited high similarity with previously reported bacterial genomes, suggesting their affiliation with known species (Fig. 2a). However, 117 MAGs shared low homology with the genomes of known species. Among these unannotated MAGs, 27 were high-quality MAGs, indicating their existence within the RM. The 552 MAGs encompassed 13 phyla, 19 classes, 51 orders, 92 families, 237 genera, and 502 species (Fig. 2b). Additionally, we identified two complete MAGs, *Fusobacterium gonidiaformans* and *Cryptobacterium curtum*, which were circularized without any gaps. The genomic sizes of these complete MAGs were 1.72 and 1.68 Mbp, respectively; 1477 and 1333 genes were annotated within their genomes (Fig. 2c, d). In addition to 116 ARGs, *F. gonidiaformans* harbored two non-ribosomal peptide synthetase-like metabolic gene clusters, suggesting capacity to assemble structurally and functionally diverse peptides with notable clinical applications (Fig. 2c). To further assess the genomic representativeness of the RGC, we performed taxonomical annotation for all enrolled samples and calculated the species frequencies among the 21 datasets (Supplementary Table 3). Of the 502 species, 112 existed in no more than five datasets, including 16 species with high-quality genomes (Fig. 1a). For each dataset, there were fewer than 500 RGC-annotated species, while there were fewer than 400 in 18 datasets, which was less than the total species number in the RGC (Fig. 1e). Based on the accumulation curves, we observed that the RGC contained sufficient species to cover the RM in the 21 datasets (Fig. 1f). Overall, these results demonstrate the representativeness of the RGC for the RM obtained from large-scale and complex metagenomic datasets.

**Figure 3:**
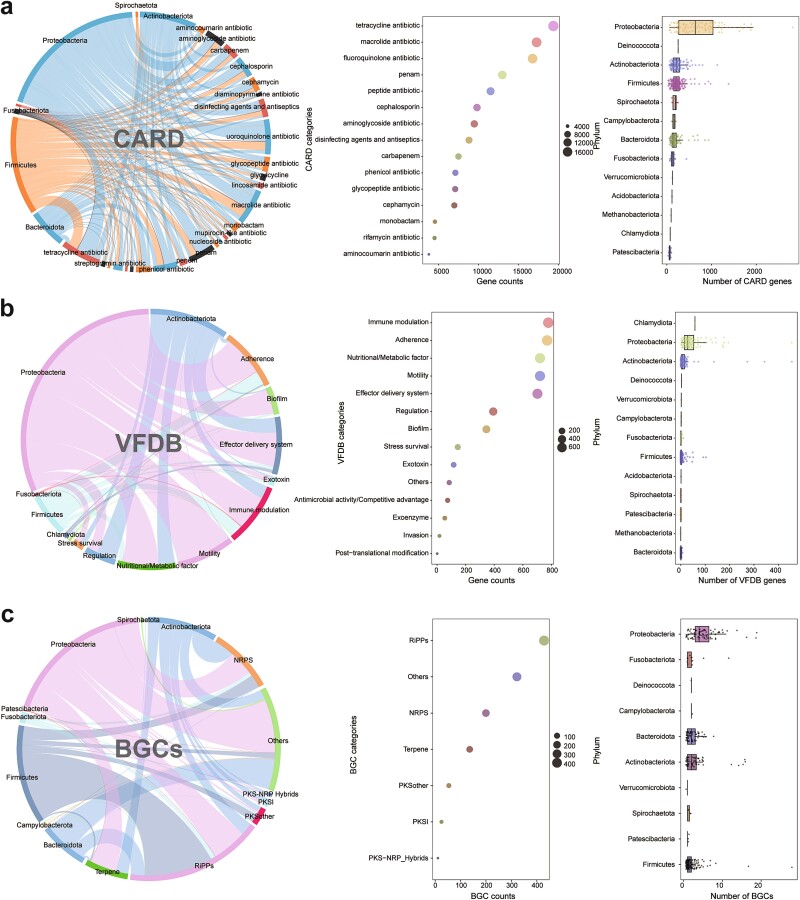
**Characteristics of the RGC genes in different functional databases.** (a–c) Annotation results of the genes predicted in the RGC compared to the CARD, VFDB, and BGC databases, respectively. Each figure presents three distinct sections from left to right: The distribution of genes from different phyla in the RGC across various functional categories in the databases, the major functional categories encompassed by the RGC, and the phyla contributing the most to the functional genes within the RGC.

The distinct functional distributions among different bacteria in the RGC enable us to gain insights into the relationship between the RM and human health. Figure 3 displays the distributions of ARGs (Fig. 3a), virulence genes (VGs) (Fig. 3b), and MGCs (Fig. 3c) in the RGC. A total of 1,092,814 genes were predicted in the RGC, including 85,391 ARGs, 8401 VGs, and 1185 MGCs (Supplementary Table 4). ARG enrichment indicated that the bacteria belonging to Proteobacteria, Actinobacteriota, and Firmicutes have resistance against tetracycline (19,341, 22.65%), macrolide (17,244, 20.19%), and fluoroquinolone (16,742, 19.61%) (Fig. 3a). Furthermore, these bacteria contained substantial VGs, particularly enriched in the categories of immune modulation (778, 9.26%), adherence (770, 9.17%), and nutritional/metabolic factors (720, 8.57%) (Fig. 3b). Among the bacteria from Proteobacteria, Actinobacteriota, Firmicutes, and Bacteroidota was a notable presence of MGCs, with enrichment in ribosomally synthesized and post-translationally modified peptides (429, 36.20%) and non-ribosomal peptide synthetase (200, 16.88%) (Fig. 3c). These findings suggest the potential of these bacteria to synthesize natural cyclic peptides with significant clinical applications. In addition, we aligned the RGC-unmapped metagenomic reads to the CARD, VFDB, and human genome and calculated their alignment ratios (Supplementary Fig. 1a). The results demonstrate that 27.5%, 41.3%, and 35.5% of the samples have RGC-unmapped reads that aligned to the CARD, VFDB, and human genome, respectively [62, 64], with only 0.101%, 0.218%, and 0.317% averaged align ratios, respectively (Supplementary Fig. 1a). To clarify the RGC-unmapped reads, we further aligned the RGC-unmapped reads to the NT and bacteriophage databases [69, 75], which contain 115,325 genomes and 873,718 phage sequences, respectively. The results suggest that the RGC-unmapped reads belonging to Bacteria, Viruses, Fungi, and Archaea took 6.913% (518,193 reads per sample in average), 0.914% (24,790 reads per sample in average), 0.081% (3541 reads per sample in average), and 0.009% (25 reads per sample in average) of the original metagenomic data, respectively (Supplementary Fig. 1b).

### Distinct microbial characteristics along different sites of the respiratory tract

**Figure 4:**
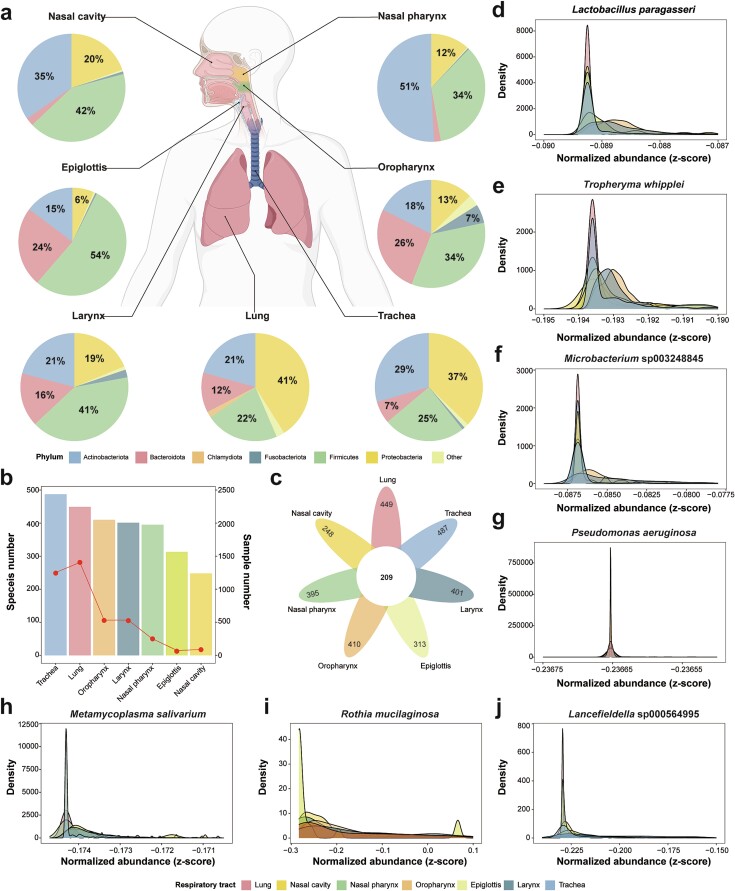
**Composition of the RM along the respiratory tract.** (a) RM compositions along the respiratory tract at the phylum level. Phylum composition was detected at seven respiratory sites. Colors indicate different phyla. (b) Species numbers in the seven respiratory sites. The x-axis represents the respiratory site. Bar height indicates species number, which corresponds to the left y-axis. The spot in the curve indicates the sample number at each respiratory site, which corresponds to the right y-axis. (c) Numbers of core species among the seven respiratory sites. The number in the center circle indicates the number of core species among respiratory sites. (d–j) Distributions of seven high-frequency and high-genome-completeness species at the seven respiratory sites.

Based on the RGC database, we detected the microbial compositions for all respiratory samples and discovered differential microbial characteristics from the upper to lower respiratory tract (Fig. 4, Supplementary Table 3). According to the collection sites, we divided the 3941 samples into seven groups: the nasal cavity, nasal pharynx, oropharynx, epiglottis, larynx, trachea, and lungs (Fig. 4a). In the nasal cavity and nasal pharynx, the dominant phyla were Firmicutes (average relative abundance: 42.07% and 34.93%, respectively), Actinobacteriota (34.70% and 50.86%, respectively), and Proteobacteria (19.54% and 11.57%, respectively). As the sampling sites approached the lower respiratory tract, we found that Bacteroidota became one of the dominant taxa (Fig. 4a). For the samples collected from the oropharynx, epiglottis, and larynx, the average relative abundance of Bacteroidota reached 26.44%, 23.93%, and 16.05%, respectively. In the lower respiratory tract, Proteobacteria became the dominant taxa; average relative abundance was 41.05% and 37.14% for the samples collected from the trachea and lungs, respectively (Fig. 4a).

Besides phyla, we detected the species compositions for the samples from the seven respiratory sites. Notably, samples from the lower respiratory tract contained the most species (Fig. 4b). For example, the samples from the lungs and nasal cavity contained 449 and 248 species, respectively. As the sampling number was higher in the lower respiratory tract (*n* = 1406 and 1243 for the lungs and trachea, respectively), we deduced that the RGC database would perform well for samples from the lower respiratory tract. In addition, seven respiratory sites shared 209 species, including *Lactobacillus paragasseri*, *Tropheryma whipplei*, and *Pseudomonas aeruginosa* (Fig. 4c). Based on the species prevalence among the 21 datasets (Fig. 1a), we further explored the distributions of seven highly prevalent species and determined their specific distribution patterns among the seven respiratory sites (Fig. 4d–j). For example, the relative abundances of *L. paragasseri* and *T. whipplei* in the lungs, nasal pharynx, and nasal pharynx were significantly lower than in the oropharynx and larynx (*P* < 0.001). Thus, these findings collectively describe the microbial features along the respiratory tract, providing a basis for taxonomical detection or comparison with the RGC database.

### Taxonomical and functional comparison between the RGC, unified human gastrointestinal genome database, and respiratory microbial gene catalogue

**Figure 5:**
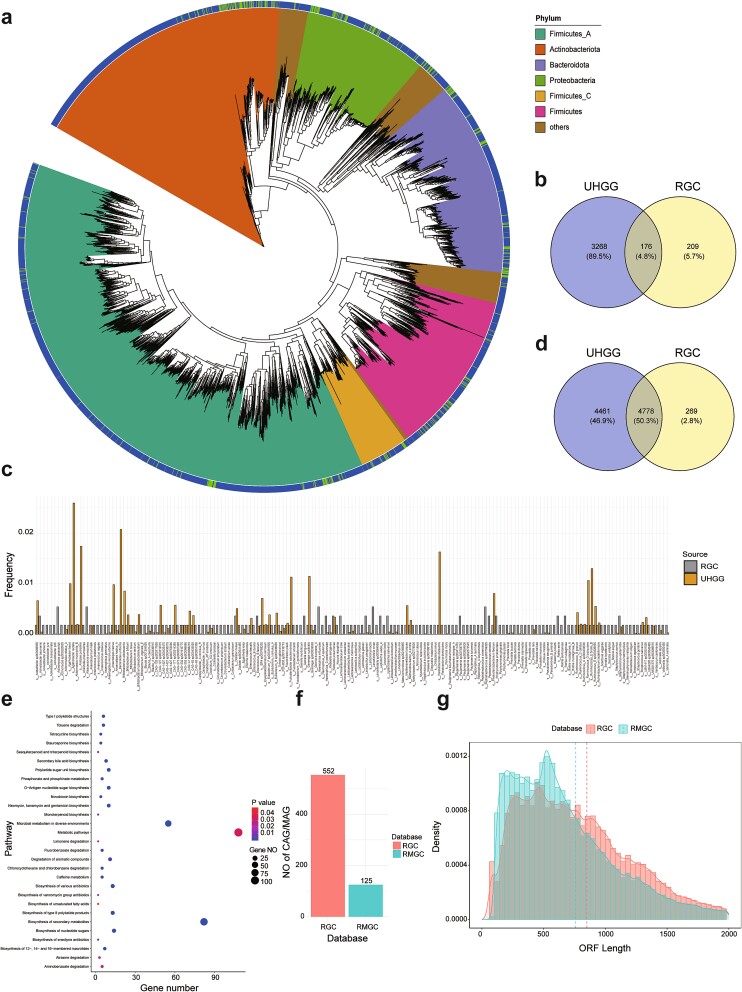
**Comparison of the RGC, UHGG, and RMGC databases.** (a) Phylogenetic tree of all RGC and UHGG genomes. The bars in outer circle indicate the genome source: The RGC and UHGG . (b) Numbers of shared and specific species between the RGC and UHGG. (c) Numbers of supporting genomes for each shared species between the RGC and UHGG. (d) Numbers of shared and specific KO identifiers between the RGC and UHGG. (e) Enriched pathways of RGC-specific KO identifiers. (f) Numbers of MAGs in the RGC and numbers of co-abundance gene groups in the RMGC. (g) Distributions of open reading frame length in the RGC and RMGC. Dashed line indicates the mean value.

The RGC includes respiratory-specific species, highlighting the inadequacy of the UHGG database for RM analysis. We applied iToL to visualize the phylogenetic tree for the 552 RGC and 4729 UHGG genomes (Fig. 5a) and discovered habitat-specific subclades in the phylogenetic tree. Then, we compared the species compositions between the RGC and UHGG (Fig. 5b), revealing that 54.3% of the species in the RGC were respiratory-specific. Among the species shared between the two databases, *Agathobacter rectalis* had the most genomes in the UHGG (Fig. 5c). In contrast, *Actinomyces graevenitzii, Alloscardovia omnicolens*, and *H. influenzae* had the most genomes in the RGC. Furthermore, the RGC and UHGG had 5047 and 9239 KEGG orthology (KO) identifiers, respectively, wherein 4778 KOs are shared between the RGC and UHGG (Fig. 5d). Moreover, functional enrichment analysis showed that 29 KEGG pathways were enriched in the RGC (*P* < 0.05, Fig. 5e).

The RGC demonstrated notable advantages in the representativeness and continuity of respiratory microbial genomes compared to the RMGC. The RGC includes 552 MAGs, whereas the RMGC only reports 125 co-abundance gene groups (Fig. 5f). Furthermore, the RGC and RMGC contain 1,122,815 and 2,245,343 non-redundant, open reading frames, respectively; the mean open reading frame lengths in the RGC and RMGC were 851 nucleotides (range: 65–43,635 nucleotides) and 761 nucleotides (range: 102–32,241 nucleotides), respectively (Fig. 5g).

### Simulated metagenomic data reveals the stable performance of the RGC

**Figure 6:**
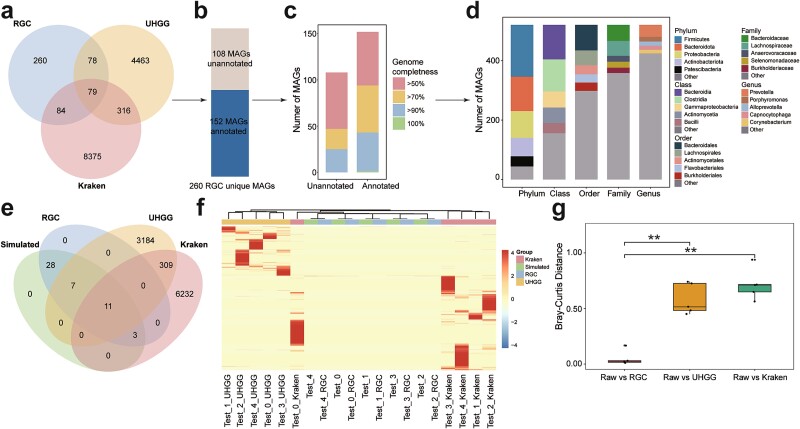
**Comparison of the RGC, UHGG, and standard database of Kraken2 for RM annotation.** (a) Numbers of species annotated using the RGC, UHGG, and standard database of Kraken2 on all enrolled metagenomic data. (b) Numbers of GTDB-annotated and GTDB-unannotated MAGs. MAGs correspond to RGC-specific species. (c) Genome completeness of the RGC-specific MAGs. (d) Taxonomic distribution of the RGC dataset at the phylum, class, order, family, and genus levels. Only the top five taxonomic units are shown for each hierarchical level, while the remaining classifications are labelled ‘other’. (e) Numbers of species annotated by the RGC, UHGG, and standard database of Kraken2 on five simulated metagenomic data. (f) Heatmap of species abundance calculated using the RGC, UHGG, and standard database of Kraken2 on five simulated metagenomic datasets. Top: Dendrograms showing the clustering results of the samples based on the Euclidean distances of species. (g) Comparison of RM similarity between the simulated and database annotated profiles. The first box indicates the Bray–Curtis distances between the simulated and RGC annotated profiles; the second box indicated the Bray-Curtis distances between the simulated and UHGG annotated profiles; and the third box indicated the Bray-Curtis distances between the simulated and standard database of Kraken2 annotated profiles (^*^^*^P < 0.01).

To examine the performance of the RGC with the RM, we conducted RM annotations for the 21 collected datasets by using the RGC, UHGG, and standard database in Kraken2. With the standard database from Kraken2, the RM samples exhibited 8854 annotated species, while the UHGG and RGC databases exhibited 4936 and 501 annotated species, respectively (Fig. 6a). The RM taxonomic profiles of these three databases shared 79 species, and the RGC annotation results included 260 specific species. We subsequently checked the taxonomical assignment and genome completeness of the MAGs corresponding to the 260 RGC-specific species (Fig. 6b, c). Among the 260 MAGs, 152 exhibited high similarity to the known bacterial genomes in the GTDB database, while 43 exhibited genome completeness >90%. On the other hand, 108 MAGs exhibited low similarity to known species, while 25 had over 90% genome completeness. In addition, the specific species annotated by the RGC were mainly from Firmicutes, Bacteroidota, Proteobacteria, and Actinobacteriota (Fig. 6d). Thus, the results suggest that compared to the UHGG and standard database in Kraken2, the RGC contains unidentified respiratory microorganisms that can deepen our understanding of the RM.

To assess the accuracy of the RGC for RM annotation, we applied the RGC, UHGG, and standard database in Kraken2 to perform taxonomical annotation and abundance calculation on five simulated respiratory metagenomic data. First, we checked the species compositions of the three databases on the simulated data (Fig. 6e). We found that the number of species annotated by the RGC was the same as that of the simulated data (*n*_species_ = 49) in contrast to the annotated species numbers from the UHGG (*n*_species_ = 3511) and standard database in Kraken2 (*n*_species_ = 6555). Second, we explored the abundance distributions of the species of the three databases using the simulated data (Fig. 6f). The results show that the species abundance calculated with the RGC was close to the simulated species abundance, whereas the species abundance calculated with the UHGG and standard database in Kraken2 poorly reflected the actual abundance in the simulated data. Third, we calculated the sample similarity between the simulated data and annotation results of the three databases (Fig. 6g). The sample similarity between the simulated data and RGC annotation results was significantly higher (Bray–Curtis distance = 0.050 ± 0.059) than that between the simulated data and UHGG (Bray–Curtis distance = 0.583 ± 0.125, *P* = 0.008) or the standard database in Kraken2 (Bray–Curtis distance = 0.716 ± 0.124, *P* = 0.008). These findings indicate that the RGC can reduce the taxonomical bias caused by non-respiratory microorganisms.

**Figure 7:**
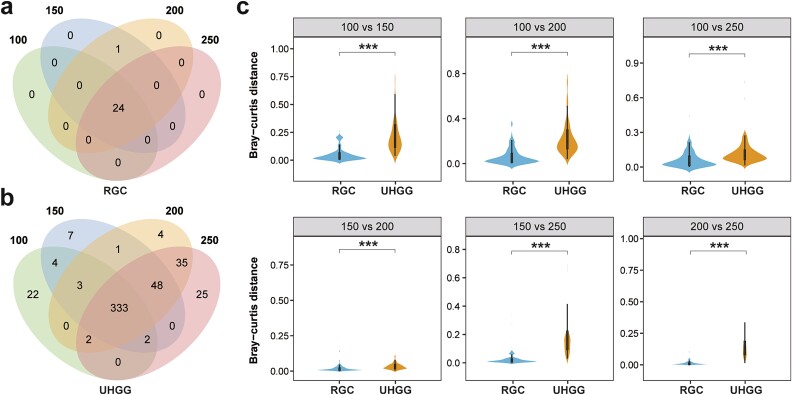
**Comparison of the annotation stability of the RGC and UHGG with metagenomic data of different read lengths.** (a) Numbers of species annotated by the RGC with metagenomic data of different read lengths. Each sample has four metagenomic datasets with different read lengths (i.e. 100, 150, 200, and 250 bp). (b) Numbers of species annotated by the UHGG with metagenomic data of different read lengths. (c) Comparison of RM similarity among datasets with different read lengths. The x- and y-axes in the plot represent the Bray-Curtis distances and the source databases, respectively (^*^^*^^*^P < 0.001).

To assess the stability of the RGC for RM annotation, we used the RGC and UHGG databases to conduct RM analysis on 88 samples, with each sample having four metagenomic data with different read lengths (i.e. 100, 150, 200, and 250 bp). First, we examined the species compositions of the RM samples under different read lengths (Fig. 7a, b). Utilizing the RGC for RM annotation, the metagenomic data with different lengths exhibited almost identical species composition, except *Lactobacillus gasseri*, which only existed in the datasets with 150 and 200 bp read lengths (Fig. 7a). However, when applying the UHGG database for RM annotation, we observed inconsistent species composition for the datasets with different read lengths (Fig. 7b). Then, we calculated the sample similarity among the datasets with different read lengths and compared them between the RGC and UHGG (Fig. 7c). Notably, the sample similarity among different read lengths was significantly lower when applying the UHGG database than the RGC database (*P* < 0.001). These findings demonstrate the stable performance of the RGC for RM taxonomical annotation with variable metagenomic read length.

### The RGC enables high-resolution analysis of respiratory metagenomic data for clinical applications

**Figure 8:**
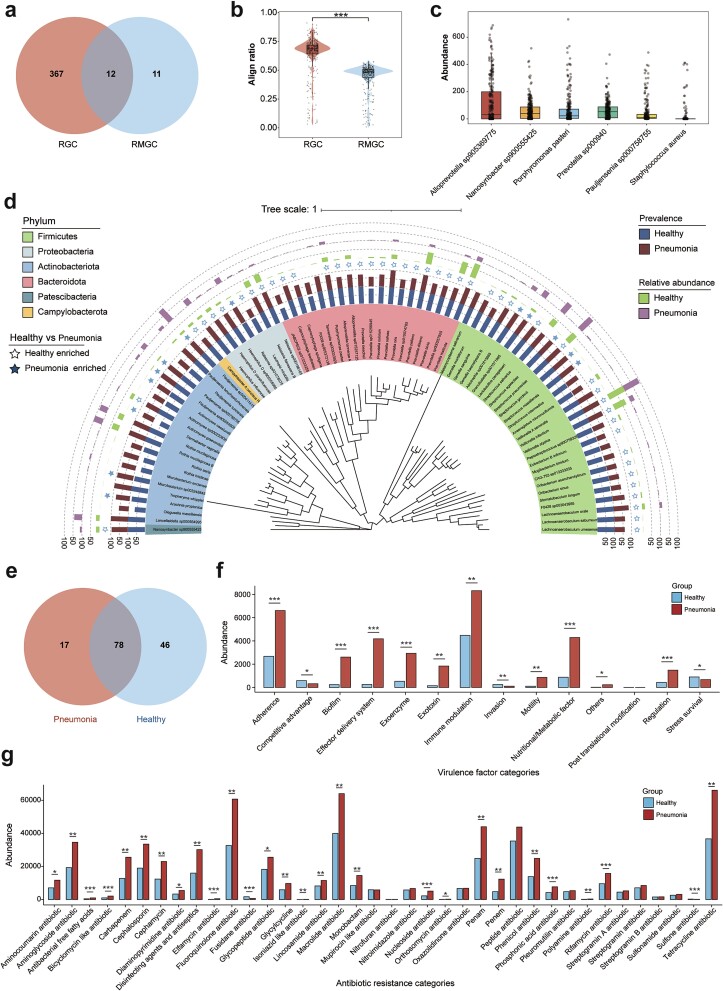
**Example practical application of the RGC with pneumonia data.** (a) Numbers of core-pan species annotated by the RGC and RMGC. (b) Alignment ratios of 334 respiratory metagenomes in the RGC and RMGC. (c) Annotation results of the RMGC-unaligned reads with the RGC database. The six most abundant species were selected from the annotation results. (d) Comparison of shared respiratory microbes between healthy children and children with pneumonia. The first (i.e. outermost) and second circles represent the relative abundance of species in the children with pneumonia and healthy children, respectively; solid stars indicate the respiratory microbes enriched in the children with pneumonia; the third and fourth circles indicate the prevalence of species in children with pneumonia and healthy children; the sixth circle represents the phylum-level classification of the species. (e) Shared and unique respiratory microbes in healthy children and children with pneumonia. (f, g) Enriched virulence factors and antibiotic resistance categories in children with pneumonia and healthy children, respectively (^*^P < 0.05, ^*^^*^P < 0.01, ^*^^*^^*^P < 0.001).

Empowered with the RGC, we re-analyzed the RM metagenomic data from 334 previously published samples and acquired RM information more comprehensive than that with the RMGC database (Supplementary Fig. 2, Supplementary Table 5) [16]. First, the RGC enables detailed taxonomical classification (Fig. 8a). Utilizing the RGC, we identified 385 distinct species from the RM of the samples, exceeding the 23 species reported in the RMGC database. Furthermore, the RGC provided detailed taxonomic classifications of these species, encompassing 16 phyla, 19 classes, 51 orders, 93 families, and 237 genera, effectively eliminating the limitation of determining the taxonomical positions for the co-abundance gene groups introduced by the RMGC (Fig. 8d, Supplementary Table 5). Second, the RGC utilized the RM metagenomic data more robustly (Fig. 8b). Among the 334 samples, an average of 63.83 ± 17.01% of the metagenomic data could be aligned to the reference genomes in the RGC, surpassing the data utilization ratio of 43.89 ± 12.45% with the RMGC (*P* < 0.001). Third, the RGC deciphered species information from the RMGC-unaligned metagenomic data (Fig. 8c). Using the RGC to analyse the metagenomic data unaligned with the RMGC, we found that these sequences were mainly attributed to *Alloprevotella* sp905369775, *Nanosynbacter* sp900555425, and *Porphyromonas pasteri*. These findings imply that the RGC provides more comprehensive RM information, overcoming the taxonomical annotation bias caused by the incompleteness of the RMGC.

Furthermore, high-resolution deciphering of the RM using the RGC assisted us in accessing the RM components and gaining deeper insights into the mechanism of the RM in pneumonia. First, the previous study inaccurately claimed the existence of opportunistic pathogens both in patients with pneumonia and healthy pediatric subjects. RGC-based analysis revealed that the oropharyngeal microbiota from children with pneumonia includes 17 unique pathogenic microorganisms, including *Staphylococcus aureus*, *Mycoplasmoides pneumoniae*, and *Staphylococcus haemolyticus* (Fig. 8e, Supplementary Table 5). However, a previous study reports these pathogens as core species in patients with pneumonia and healthy pediatric subjects, contradicting the sample grouping information. Second, the RGC unveiled distinct RM characteristics in children with pneumonia that were not identified in previous studies. For the 78 shared microorganisms between children with pneumonia and healthy children, those with pneumonia exhibited significantly higher abundance and prevalence of *Rothia mucilaginosa* (*P*_adj_ < 0.001) and *Ralstonia sp001078575* (*P*_adj_ < 0.001) (Fig. 8d, Supplementary Table 6). Conversely, the abundance and prevalence of *Veillonella infantium* (*P*_adj_ < 0.001) and *Prevotella histicola* (*P*_adj_ < 0.001) were significantly lower in children with pneumonia (Fig. 8d, Supplementary Table 6). Third, the RGC comprehensively illustrated the distribution of the VGs and ARGs in the RM of children with pneumonia. With the enrichment of opportunistic pathogens, the RM of children with pneumonia contained higher abundances of VGs, notably enriched in adherence (*P*_adj_ < 0.001), effector delivery system (*P*_adj_ < 0.001), and immune modulation (*P*_adj_ = 0.007) (Fig. 8f, Supplementary Table 7). Furthermore, the RM of children with pneumonia exhibited greater antibiotic resistance, including resistance to macrolide (*P*_adj_ = 0.002), fluoroquinolone (*P*_adj_ = 0.002), and tetracycline (*P*_adj_ = 0.003) (Fig. 8g, Supplementary Table 7).

### The RGC contributes to the rapid and specific diagnosis of respiratory infections

**Figure 9:**
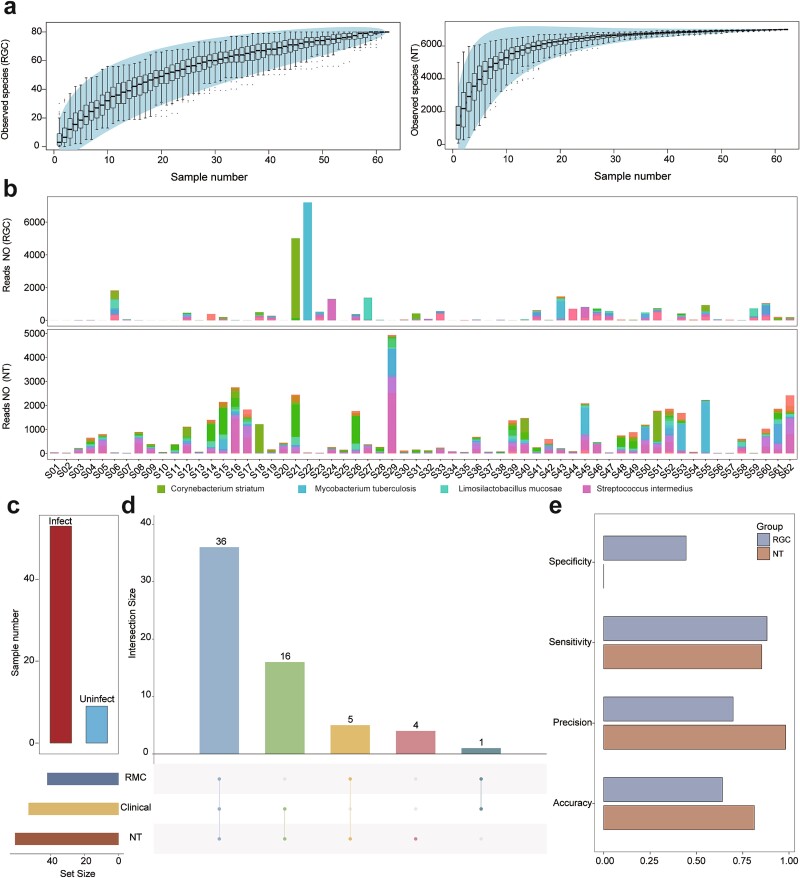
**Assessment of respiratory infections in 62 participants using the RGC.** (a) Cumulative curves derived from the microbial annotation of BALF samples from 62 participants using the RGC and NT databases. Left and right panels display the cumulative curves for the RGC and NT annotations, respectively. (b) Bacterial annotations of BALF samples from 62 participants using the RGC and NT databases. Bar length corresponds to species abundance, and colors represent different species. (c) Numbers of infected and uninfected participants after final clinical diagnosis. (d) Upset graph illustrating the concordance between the RGC annotation results, NT annotation results, pathogenic culture results, and final clinical diagnoses. (e) Venn diagram of the numbers of common and unique species among the 62 BALF samples after annotation using the RGC and NT databases. The bar plot shows the accuracy, precision, sensitivity, and specificity of clinical infection diagnosis based on the RGC and NT databases.

Compared to the NT, the RGC demonstrates superior sensitivity and specificity for the diagnosis of respiratory infections. To assess the clinical utility of the RGC, we applied it to clinical samples for respiratory infection diagnosis. We submitted the BALF samples from 62 patients to RM annotation and respiratory infection diagnosis using the RGC and NT databases (Supplementary Table 8). Then, we committed the inferred results to the final clinical diagnosis to evaluate concordance, and discovered that the RGC effectively mitigated the substantial noise signals introduced by the NT database, enhancing the diagnostic efficacy of respiratory infections. Among the 62 BALF samples, the RGC annotated 79 bacterial species, while the NT database identified 7008 bacterial species (Fig. 9a, Supplementary Fig. 3). Despite the significant disparity in the number of respiratory species identified by the two databases, the RGC captured a comprehensive representation of the RM in the participants (Fig. 9a).

Furthermore, owing to the diminished noise signals, the RGC facilitated the rapid identification of the predominant respiratory pathogens, such as *Mycobacterium tuberculosis* and *Limosilactobacillus mucosae*, which would enable clinicians to rapidly determine the specific infection types (Fig. 9b). Additionally, the RGC enhanced the sensitivity and specificity of respiratory infection diagnosis (Fig. 9c–e). We compared the inferred results from the RGC and NT databases with the final clinical diagnosis to evaluate their concordance (Supplementary Table 9). The diagnostic results of 37 cases using the RGC were consistent with the final clinical diagnosis, while the NT database showed concordance in 52 cases (Fig. 9d). The higher diagnostic accuracy of the NT database may be due to the sample bias in the studied population (*n*_Infect_ = 53, Fig. 9c). Nevertheless, the RGC demonstrated superior performance to the NT in other clinical diagnostic indices, including specificity (0.444 versus 0, respectively) and sensitivity increased (0.881 versus 0.852, respectively) (Fig. 9e).

## Discussion

In this study, we constructed an RGC by employing long-read Nanopore and short-read next-generation sequencing data derived from 4191 respiratory samples. The RGC consists of 522 genomes that fulfil the median criteria of the MIMAG standards [22]. Including such a diverse array of sample sources enhances the richness and representativeness of the RGC, thereby making it a valuable reference for exploring the composition of the RM. Furthermore, to our knowledge, the RGC documents the largest collection of respiratory microbial genomes, being more than four times larger than the previous RMGC. Consequently, the RGC is an invaluable resource that paves the way for further research on the RM.

Here, we developed an optimizing strategy to advance the continuity of metagenomic assemblies. By leveraging the conjugate graph concept, we can efficiently model the complex double-helical structure of DNA, capturing critical information to produce accurate contiguous assemblies. Furthermore, by modelling the scaffolding process, finding the weighted maximum bipartite matching problem on the conjugate graph and solving it using a constrained Kuhn–Munkres algorithm, our method effectively resolves the orders and directions of contigs. This optimization strategy improves the continuity, accuracy, and resolution of the MAGs obtained directly from complex next-generation sequencing metagenomic data. Using this method, we developed the RGC, an extensive collection of high-quality respiratory genomes. In particular, the RGC demonstrates substantial advances in the continuity and representativeness of respiratory microbial genomes compared to the previously constructed RMGC. With a diverse range of sample sources, the RGC includes 522 high-quality respiratory microbial genomes (4.4 times more than the RMGC), highlighting its representativeness for the microbes in the RM. Additionally, the RGC features the longest contig spanning 1.72 Mbp (a complete MAG) and an N50 of 13,173 bp, significantly exceeding the RMGC’s N50 of 981 bp and underscoring the novel genome continuity achieved by the RGC. Overall, the RGC contains comprehensive and high-quality respiratory microbial genomes, serving as a fundamental resource for establishing standardized repositories of respiratory genomes and further facilitating metagenomic investigation of the RM.

When comparing the RGC and UHGG, we observed that the RM has specific subclades in the genome compared to the gut microbiome. Only 45.7% of RM species are shared between the respiratory and digestive tracts. However, 94.7% of the RGC KO identifiers were detected in the gut microbiome. The respiratory-specific KOs are enriched in pathways of the biosynthesis of secondary metabolites (*P* < 0.001) and the biosynthesis of type II polyketide products. These pathways might play vital roles in microbial adaptation in the human respiratory tract. The respiratory tract contains a relatively large number of specific species but few specific genes compared to the RMGC. This might be because the RMGC integrates publicly available genes from the Human Microbiome Project, the Pathosystems Resource Integration Center, and the Integrated Microbial Genomes and Microbiomes, which include a substantial number of non-respiratory microbial genes.

Additionally, this study demonstrated the accuracy and stability of the RGC for RM annotation. Using the simulated metagenomic data, the RM results from the RGC exhibited the same species composition and species abundance close to the simulated data compared to the RM annotations from the UHGG database or the standard database in Kraken2. This, the results not only demonstrate the accuracy of the RGC for RM annotation, but also show that it reduces the taxonomical bias caused by non-respiratory microorganisms. Furthermore, compared to the UHGG database, the RGC yielded consistent species composition and abundance for the metagenomic data from the same samples with varying read lengths. Thus, the results collectively demonstrate the stable performance of the RGC for RM taxonomical annotation using metagenomic data with either long or short read length.

We also assessed the clinical utility of the RGC using the metagenomic data from a previous pneumonia study, offering a detailed etiological characterization for the children with pneumonia [16]. The diversity of the RM is potentially crucial for human health [76]. The RM of children with pneumonia exhibits reduced species diversity compared to that in healthy children, indicating a simplified microbial community structure resulting from intense immune responses in patients [77]. In addition, the RGC identified 385 respiratory microbial species across all samples, surpassing the 125 species annotated in previous studies and highlighting the superiority of the RGC in capturing the RM spectrum. Furthermore, the RGC confirmed the presence of 17 pneumonia-specific respiratory pathogens, including *Staphylococcus aureus*, *Mycoplasma pneumoniae*, and *S. haemolyticus*. These pathogens are reported to be present in both children with pneumonia and healthy children [16], suggesting potential etiological judgment errors due to an incomplete RM database, which would have impeded a deeper understanding of the RM.

Furthermore, we showed that the RGC demonstrates superior sensitivity and specificity for diagnosing respiratory infections. Compared to the NT database, the RGC had fewer microbial annotations, effectively mitigating the substantial noise signals introduced by non-respiratory microorganisms present in the NT database. Furthermore, the RGC showed better sensitivity and specificity for respiratory infection diagnosis than the public database. These findings underscore the significance of a complete and representative RGC in facilitating examination of the RM, which is crucial for diagnosing respiratory infections.

Nevertheless, one limitation of this study is the inadequate recovery of fungi, archaea, and viruses in the RGC. As bacteria account for up to 98% of the genetic material in metagenomic samples, other microorganisms, such as fungi, archaea, and viruses, were captured incompletely in the RGC, leading to small fungal, archaeal, and viral genomic fragments during assembling [78], which were filtered from the RGC (shorter than 1000 bp). In addition, the vast diversity of virus types and the high genome similarity between the bacteriophages and the bacteria make it more difficult to isolate viruses from metagenomes [79]. Another limitation is the limited number of complete MAGs. Owing to the insufficient respiratory metagenomic data, which is hindered by host genomic data, it is difficult to recover complete MAGs. However, future advances in low-input, high-depth sequencing technologies will offer opportunities to obtain more complete MAGs for RM.

In summary, we constructed a non-redundant, highly contiguous, and representative RGC based on the large-scale integration of second- and third-generation metagenomic data from respiratory samples. This invaluable resource will provide fundamental support for researchers and clinicians to access precise information on the RM composition, functional features, and clinical diagnosis.

**Key Points**

- The respiratory genome catalogue (RGC) presents highly contiguous and representative microbial genomes in the human respiratory tract.
- The RGC contains respiratory-specific species, enabling high-resolution and precision identification of the respiratory microbiome.
- The RGC exhibits superior sensitivity and specificity for the diagnosis of respiratory infections compared to other databases.

## Supplementary Material

Supplementary_Figures_bbae620

Supplementary_Tables_bbae620

## Data Availability

The comprehensive human RGC and related information are publicly available in the Zenodo repository (https://zenodo.org/record/8103433). The code for the metagenomic data simulation is publicly available at GitHub (https://github.com/wshuai294/RGC_analysis). Other data underlying this article are available in the article and in its Online Supplementary Material.
