# Supplementary figures and images for: Comprehensive human respiratory genome catalogue underlies the high resolution and precision of the respiratory microbiome
Source: Brief Bioinform. 2024 Nov 24;26(1):bbae620. doi: 10.1093/bib/bbae620 (PMC11586125; doi:10.1093/bib/bbae620)

# Supplementary Figure 1

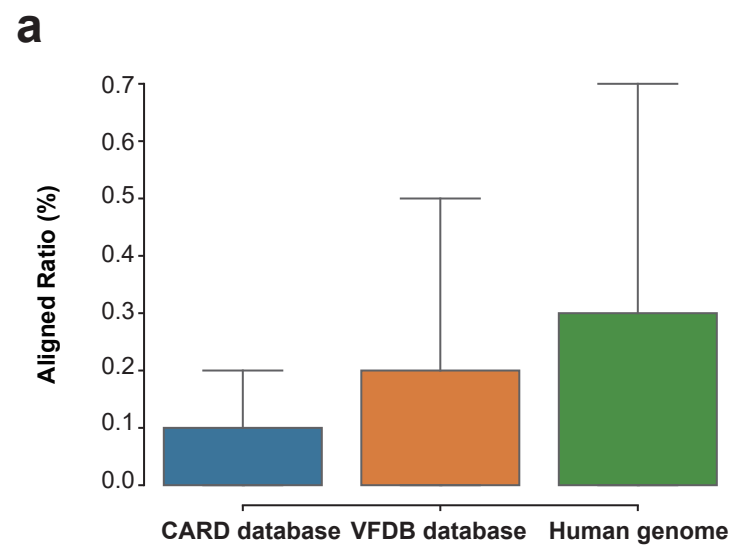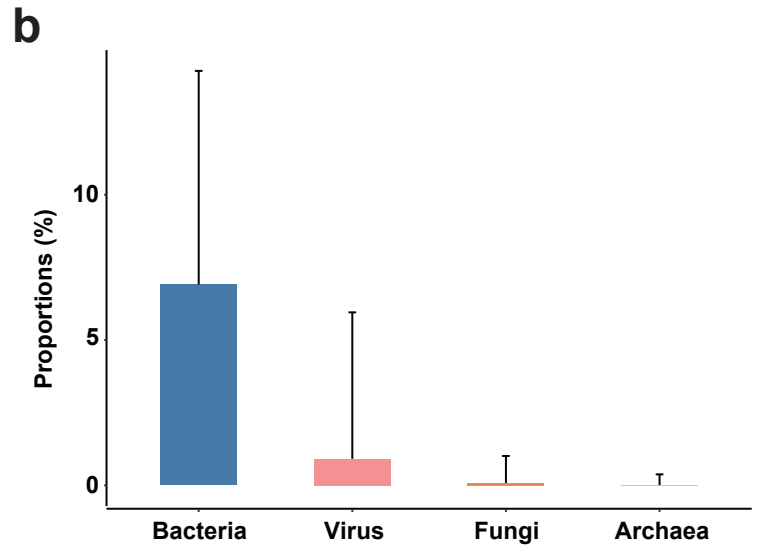

# Supplementary Figure 2

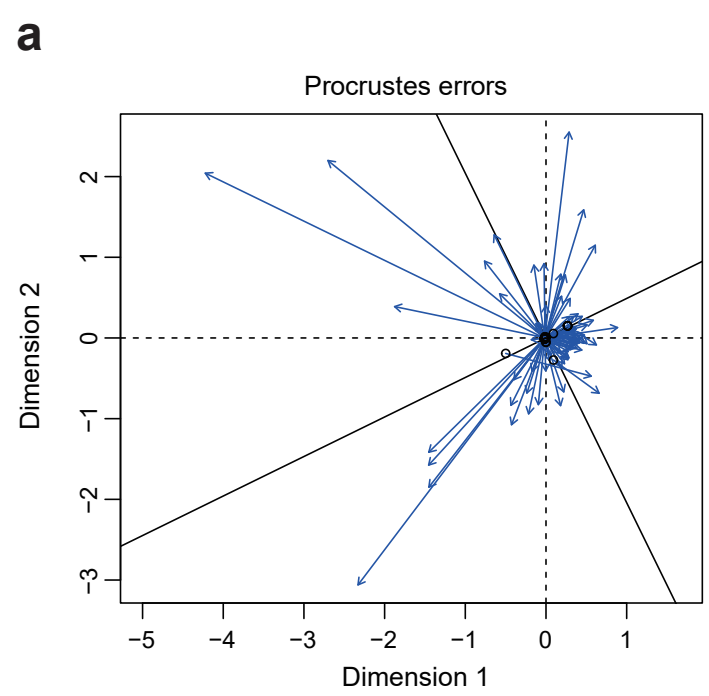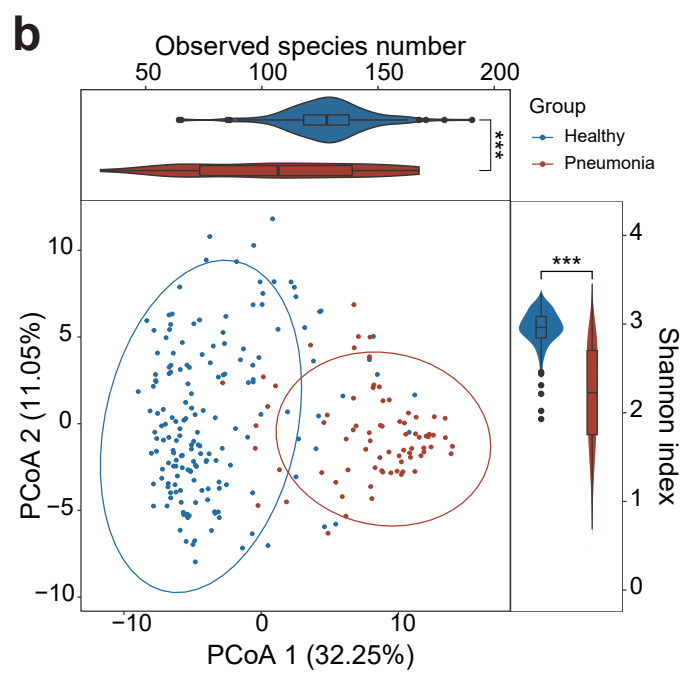

# Supplementary Figure 3

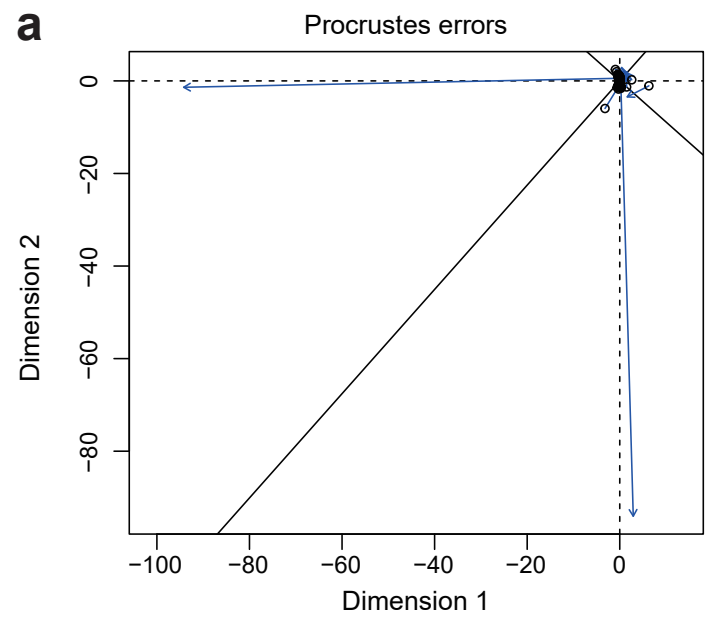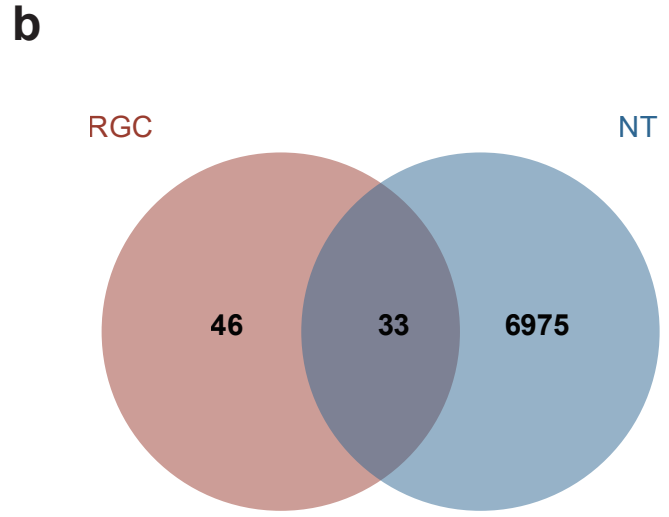

Supplement: Supplementary_Figures_bbae620 [file supplementary_figures_bbae620.pdf]
